# In the context of a case: Aripiprazole to resume habits

**DOI:** 10.1192/j.eurpsy.2025.1066

**Published:** 2025-08-26

**Authors:** A. B. Mulero García, E. S. Martinez, C. Lopez Morillo, G. Perez Guerrero

**Affiliations:** 1 Hospital Regional Universitario de Málaga, Málaga, Spain

## Abstract

**Introduction:**

A 43-year-old male with a diagnosis of Bipolar Disorder associated with substance use is admitted to the mental health unit due to a psychotic decompensation. The patient has had an unfavorable progression requiring multiple admissions to mental health services. He suffers from dyslipidemia, hypertension, and poorly controlled diabetes. He has had some occasional work but is currently unemployed. He does not engage in leisure or sports activities, which he attributes to being highly sedated by the medication. He is currently being treated with clozapine, valproic acid, quetiapine, and various benzodiazepines.

**Objectives:**

Achieve stabilization of the psychopathological condition without producing additional side effects such as sedation.

**Methods:**

During the hospital stay, Quetiapina is slowly reduced over one week, and Aripiprazole is introduced at a moderate oral dose (Aripiprazole 10 mg). It is then gradually increased to 30 mg/day. After 3 weeks, it is switched to Aripiprazole 400 mg /28 days intramuscularly. At the start of Aripiprazole introduction, there is a good response and a tolerance.

With the combination of Clozapine and Aripirazole 400 mg/28 days intramuscularly, there is a noticeable reduction in psychotic symptoms, allowing for the discontinuation of both Quetiapine and benzodiazepines from the patient’s treatment.

**Results:**

At present, reviewing the outpatient follow-ups the patient has had after hospital discharge, he continues with Aripiprazole 400 mg /28 days intramuscularly, showing overall improvement in functioning. Due to the reduction in sedation, the patient has begun participating in sports and educational activities, which provide him significant personal satisfaction.

**Conclusions:**

Introducing Aripiprazole in combination with Clozapine significantly reduces psychotic symptoms by controlling positive symptoms, which means the patient does not need to rely on benzodiazepines, allowing for their discontinuation. The reduced level of sedation has enabled the patient to engage in sports and other activities that he previously felt hindered from due to medication. It is indeed very important for any patient to resume leisure and sports activities, but in this specific case, the patient had grade III obesity with associated dyslipidemia.

**Disclosure of Interest:**

None Declared

